# Genome-wide classification and expression analysis of *MYB* transcription factor families in rice and Arabidopsis

**DOI:** 10.1186/1471-2164-13-544

**Published:** 2012-10-10

**Authors:** Amit Katiyar, Shuchi Smita, Sangram Keshari Lenka, Ravi Rajwanshi, Viswanathan Chinnusamy, Kailash Chander Bansal

**Affiliations:** 1 National Research Centre on Plant Biotechnology, Indian Agricultural Research Institute, New Delhi, 110012, India; 2 National Bureau of Plant Genetic Resources, Indian Agricultural Research Institute Campus, New Delhi, 110012, India; 3 Department of Biology, University of Massachusetts, Amherst, MA, 01003, USA; 4 Department of Biotechnology, Assam University, Silchar, Assam, 788011, India; 5 Division of Plant Physiology, Indian Agricultural Research Institute, New Delhi, 110012, India

## Abstract

**Background:**

The *MYB* gene family comprises one of the richest groups of transcription factors in plants. Plant MYB proteins are characterized by a highly conserved MYB DNA-binding domain. MYB proteins are classified into four major groups namely, 1R-MYB, 2R-MYB, 3R-MYB and 4R-MYB based on the number and position of MYB repeats. *MYB* transcription factors are involved in plant development, secondary metabolism, hormone signal transduction, disease resistance and abiotic stress tolerance. A comparative analysis of *MYB* family genes in rice and Arabidopsis will help reveal the evolution and function of *MYB* genes in plants.

**Results:**

A genome-wide analysis identified at least 155 and 197 *MYB* genes in rice and Arabidopsis, respectively. Gene structure analysis revealed that *MYB* family genes possess relatively more number of introns in the middle as compared with C- and N-terminal regions of the predicted genes. Intronless *MYB*-genes are highly conserved both in rice and Arabidopsis. *MYB* genes encoding R2R3 repeat MYB proteins retained conserved gene structure with three exons and two introns, whereas genes encoding R1R2R3 repeat containing proteins consist of six exons and five introns. The splicing pattern is similar among R1R2R3 *MYB* genes in Arabidopsis. In contrast, variation in splicing pattern was observed among R1R2R3 *MYB* members of rice. Consensus motif analysis of 1kb upstream region (5′ to translation initiation codon) of *MYB* gene ORFs led to the identification of conserved and over-represented *cis*-motifs in both rice and Arabidopsis. Real-time quantitative RT-PCR analysis showed that several members of *MYBs* are up-regulated by various abiotic stresses both in rice and Arabidopsis.

**Conclusion:**

A comprehensive genome-wide analysis of chromosomal distribution, tandem repeats and phylogenetic relationship of *MYB* family genes in rice and Arabidopsis suggested their evolution *via* duplication. Genome-wide comparative analysis of *MYB* genes and their expression analysis identified several *MYBs* with potential role in development and stress response of plants.

## Background

Transcription factors are essential regulators of gene transcription and usually consist of at least two domains namely a DNA-binding and an activation/repression domain, that function together to regulate the target gene expression [1]. The *MYB* (myeloblastosis) transcription factor family is present in all eukaryotes. "Oncogene" *vMYB* was the first *MYB* gene identified in avian myeloblastosis virus [2]. Three *vMYB*-related genes namely c-*MYB*, A-*MYB* and B-*MYB* were subsequently identified in many vertebrates and implicated in the regulation of cell proliferation, differentiation, and apoptosis [3]. Homologous genes were also identified in insects, fungi and slime molds [4]. A homolog of mammalian c-*MYB* gene, *Zea mays C1*, involved in regulation of anthocyanin biosynthesis, was the first *MYB* gene to be characterized in plants [5]. Interestingly, plants encode large number of *MYB* genes as compared to fungi and animals [6-12]. MYB proteins contain a MYB DNA-binding domain, which is approximately 52 amino acid residues in length, and forms a helix-turn-helix fold with three regularly spaced tryptophan residues [13]. The three-dimensional structure of the MYB domain showed that the DNA recognition site α-helix interacts with the major groove of DNA [14]. However, amino acid sequences outside the MYB domain are highly divergent. Based on the number of adjacent MYB repeats, *MYB* transcription factors are classified into four major groups, namely 1R-MYB, 2R-MYB, 3R-MYB and 4R-MYB containing one, two, three and four MYB repeats, respectively. In animals, R1R2R3-type MYB domain proteins are predominant, while in plants, the R2R3-type MYB domain proteins are more prevalent [4,7,15]. The plant R2R3-*MYB* genes probably evolved from an R1R2R3-*MYB* gene progenitor through loss of R1 repeat or from an *R1MYB* gene through duplication of R1 repeat [16,17].

In plants, *MYB* transcription factors play a key role in plant development, secondary metabolism, hormone signal transduction, disease resistance and abiotic stress tolerance [18,19]. Several R2R3-*MYB* genes are involved in regulating responses to environmental stresses such as drought, salt, and cold [9,20]. Transgenic rice over expressing *OsMYB3R2* exhibited enhanced cold tolerance as well as increased cell mitotic index [21]. Enhanced freezing stress tolerance was observed in Arabidopsis over-expressing *OsMYB4*[10,22]. Arabidopsis *AtMYB96*, an R2R3-type *MYB* transcription factor, regulates drought stress response by integrating ABA and auxin signals [23]. Transgenic Arabidopsis expressing *AtMYB15* exhibited hypersensitivity to exogenous ABA and improved tolerance to drought [24], and cold stress [20]. The *AtMYB15* negatively regulated the expression of *CBF* genes and conferred freezing tolerance in Arabidopsis [20]. Other functions of MYBs include control of cellular morphogenesis, regulation of secondary metabolism, meristem formation and the cell cycle regulation [15,25-28]. Recent studies have shown that the *MYB* genes are post-transcriptionally regulated by microRNAs; for instance, *AtMYB33*, *AtMYB35*, *AtMYB65* and *AtMYB101* genes involved in anther or pollen development are targeted by miR159 family [29,30].

*MYB* TF family genes have been identified in a number of monocot and dicot plants [9], and evolutionary relationship between rice and Arabidopsis MYB proteins has been reported [31]. We report here genome-wide classification of 155 and 197 *MYB* TF family genes in rice and Arabidopsis, respectively. We also analysed abiotic stress responsive and tissue specific expression pattern of the selected *MYB* genes. To map the evolutionary relationship among *MYB* family members, phylogenetic trees were constructed for both rice and Arabidopsis MYB proteins. Several over- represented *cis*-regulatory motifs in the promoter region of the *MYB* genes were also identified.

## Results and discussion

### Identification, classification and structural analysis of MYB family members

**Figure 1:**
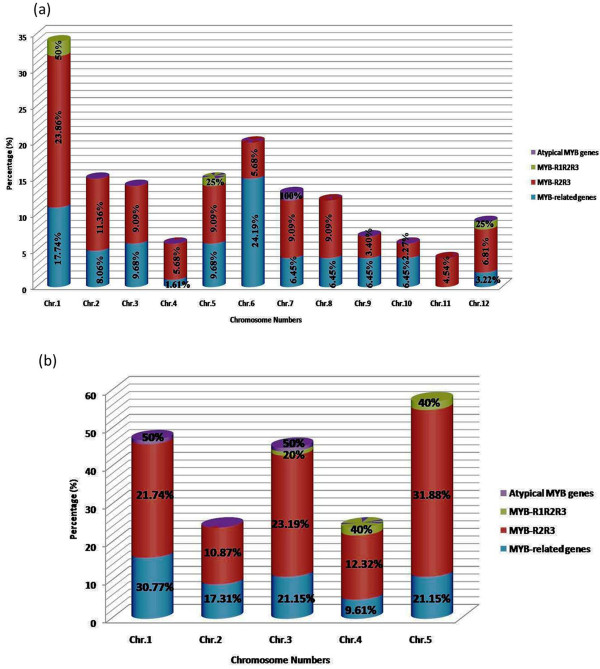
**Chromosome-wise distribution of *MYB* transcription factor genes. a**) rice, **b**) Arabidopsis. We classified *MYB* transcription factors in to four distinct groups namely “MYB-related genes”, “MYB-R2R3”, “MYB-R1R2R3”, and “Atypical MYB genes” based on the presence of one, two, three and four MYB repeats, respectively.

Genome-wide analysis led to the identification of 155 and 197 *MYB* genes in rice and Arabidopsis, respectively, with their mapping on different chromosomes (Additional file 1: Table S1). We used previously assigned names to the *MYB* genes; for instance, *AtMYB0* (*GL1*) name was accepted for the first identified R2R3 *MYB* gene; subsequently identified R2R3 *MYB* genes were named as *AtMYB1*, *AtMYB2*, etc. in Arabidopsis [31-34]. We classified *MYB* transcription factors in to four distinct groups namely “MYB-related genes”, “MYB-R2R3”, “MYB-R1R2R3”, and “Atypical MYB genes” based on the presence of one, two, three and four MYB repeats, respectively. Our analysis revealed that the MYB-R2R3 subfamily consisted of the highest number of *MYB* genes, with 56.77 and 70.05% of the total *MYB* genes in rice and Arabidopsis, respectively (Figure 1a, b). In the R2R3-MYB proteins, N-terminal consists of MYB domains, while the regulatory C-terminal region is highly variable. Presence of a single MYB-like domain (e.g. hTRF1/hTRF2) in their C terminus is required for telomeric DNA binding *in vitro*[35]. Earlier study revealed that the R2R3-MYB related proteins arose after loss of the sequences encoding R1 in an ancestral 3R-*MYB* gene during plant evolution [36]. In contrast, only few *MYB*-R1R2R3 genes were identified in Arabidopsis and rice with 5 and 4 genes, respectively. The category “MYB-related genes” usually but not always contain a single MYB domain [17,31,36]. We found that “MYB-related genes” represented 40 and 26.39% of the total *MYB* genes in rice and Arabidopsis, respectively (Figure 1a, b), and thus constituted the second largest group of MYB proteins in both rice and Arabidopsis. We also identified one MYB protein in rice and two MYB proteins in Arabidopsis that contained more than three MYB repeats and these belong to “Atypical MYB genes” group. The AT1G09770 in Arabidopsis and LOC_Os07g04700 in rice have five MYB domains and are called as CDC5-type protein, whereas AT3G18100 of Arabidopsis has four MYB domains and is named as 4R-type MYB (Table 1; Additional file 1: Table S1). The 4R-MYB proteins belong to the smallest class, which contains R1/R2-like repeats. *MYB* genes can also be classified into several subgroups based on gene function, such as Circadian Clock Associated1 (CCA1) and Late Elongated Hypocotyl (LHY), Triptychon (TRY) and Caprice (CPC) [15,17,37]. CPC and TRY belong to the R3-MYB group and are mainly involved in epidermal cell differentiation, together with *ENHANCER OF TRY AND CPC1*, *2* and *3* (*ETC1*, *ETC2* and *ETC3*), and *TRICHOMELESS1* and *2* (*TCL1 and TCL2*) [38-41]. Here, we observed that CCA1, CPC and LHY subgroups contain 23, 3 and 1 ‘MYB-related’ TF, respectively in Arabidopsis. To further understand the nature of MYB proteins, their physiochemical properties were also analyzed. The MYB proteins have similar grand average hydropathy (GRAVY) scores. Kyte and Doolittle [42] proposed that higher average hydropathy score of a protein indicates physiochemical property of an integral membrane protein, while a negative score indicates soluble nature of the protein. We observed that all MYB proteins in rice and Arabidopsis, except AT1G35516 had a negative GRAVY score, suggesting that MYBs are soluble proteins, a character that is necessary for transcription factors. Minimum and maximum score of GRAVY were recorded as −1.287 (LOC_Os02g47744) and −0.178 (LOC_Os08g37970) in rice, and −1.359 (AT5G41020) and 0.612 (AT1G35516) in Arabidopsis, respectively. We also calculated average isoelectric point (pI) value. The mean pI values for MYB-1R, R2R3 and R1R2R3 protein families were 7.55, 6.90 and 7.25 in rice, and 7.55, 6.89 and 6.80 in Arabidopsis, respectively. The average molecular weight of MYB-1R, R2R3 and R1R2R3 protein families were 31.128, 34.561 and 72.52 kDa in rice, and 34.186, 35.875 and 86.217 kDa in Arabidopsis, respectively (Additional file 1: Table S1).

**Table 1:** **MYB**-**domain based characterization and comparison of *MYB *transcription factor family genes in terms of GRAVY**, **molecular weight and cellular localization**

**RICE**												
**MYB groups**	**No of genes**	**(%)**	**GRAVY**			**PI**			**Molecular weight**			**Localization**
			**Min.**	**Max.**	**Avg.**	**Min.**	**Max.**	**Avg.**	**Min.**	**Max.**	**Avg.**	
MYB-related genes	62	40	−1.287	−0.201	−1.3875	3.99	12.26	8.125	7613.7	170921.8	89267.75	Nuclear
MYB-R2R3	88	56.77	−0.906	−0.178	−0.995	4.67	10.4	7.535	21605.3	75878.9	48742.1	Nuclear
MYB-R1R2R3	4	2.58	−0.691	−0.593	−0.9875	5.05	8.53	13.605	64100.1	109413.5	86756.8	Nuclear
Atypical MYB genes	1	0.64	−0.748	−0.748	−0.748	9.56	9.56	9.56	92424.6	92424.6	92424.6	Nuclear
**ARABIDOPSIS**												
**MYB groups**	**No of genes**	**(%)**	**GRAVY**			**PI**			**Molecular weight**			**Localization**
			**Min.**	**Max.**	**Avg.**	**Min.**	**Max.**	**Avg.**	**Min.**	**Max.**	**Avg.**	
MYB-related genes	52	26.39	−1.359	0.612	−0.3735	4.75	6.62	2.375	7570.9	50112	3785.45	Nuclear
MYB-R2R3	138	70.05	−1.102	−0.471	−0.7865	4.16	10.24	7.2	27951.2	33239	13975.6	Nuclear
MYB-R1R2R3	5	2.54	−0.941	−0.774	−0.8575	5.43	9.22	7.325	50032.2	158268.4	79134.2	Nuclear
Atypical MYB genes	2	0.51	−0.941	−0.94	−0.9405	5.67	6.37	3.185	95766.5	96084.3	95925.4	Nuclear

### Functional classification of *MYB* transcription factors

MYB proteins perform wide diversity of functions in plants. The R2R3-MYB proteins are involved in plant specific processes, such as control of secondary metabolism or cellular morphogenesis [43-49]. Gene ontology (GO) analysis suggested that R2R3-*MYB* genes, namely *AtMYB16*, *AtMYB35*, *AtMYB5*/*AtMYB80*, and *AtMYB91* may regulate cell, anther, trichome and leaf morphogenesis, respectively. Likewise, R2R3-type genes, namely *OsMYB16*, *OsMYB88*, *OsMYB117*, LOC_Os01g50110 and LOC_Os03g38210 may regulate morphogenesis in rice. In addition to R2R3-type *MYBs*, two *MYB*-related genes, LOC_Os01g43180 and LOC_Os09g23200 may also regulate morphogenesis in rice. R2R3-type *AtMYB10* and AT2G47210, *MYB*-related AT3G09600, and R1R2R3-type *AtMYB3R4* genes were identified with GO function, such as N-terminal protein myristoylation, histone H3 acetylation, and regulation of DNA endoreduplication, respectively. Previous studies have shown that genes encoding 3R-MYB proteins have regulatory role in cell cycle control [28,50]. We also found that *AtMYB3R4* may be involved in cell cycle control (GO: 0007049). GO analysis of MYB proteins illustrated that 98.70% *OsMYB* and 98.47% *AtMYB* were fully involved in transcription activation, while rest of the MYB proteins were classified in to other GO functions, such as kinase activity, protein binding, transcription repressor activity, etc. GO analysis categorized rice LOC_Os01g62660 as signal transducer (GO: 0004871) and transcription activator. The R2R3-type *AtMYB4* was classified into transcriptional repressor group. The *AtMYB4* expression is down regulated by exposure to UV-B light, indicating that derepression of its target genes is an important mechanism for acclimation to UV-B in Arabidopsis [51,52]. In our study, *AtMYB34*; a R2R3-type MYB protein, has been found with catalytic-kinase as well as transcription activator molecular functions as reported earlier [53,54]. The *AtMYB34* is also involved in defense response against insects [55]. In consistent with previous report [56], *AtMYB23* was found to have protein binding (i.e. interaction with GL3) as well as DNA-binding functions.

The subcellular localization of MYB proteins was predicted using several localization predictor softwares. The predicted locations of the MYB proteins were also verified by gene ontology under keyword “GO cellular component” and species-specific localization prediction tools, e.g., AtSubP for Arabidopsis [57] to enhance the accuracy of prediction. Consensus outcome revealed that 98.71% OsMYB and all AtMYB proteins were found to be nuclear localized and confirmed by the presence of nuclear localization signal (NLS). The remaining two members of MYB proteins in rice were predicted to be localized in mitochondria and plasma membrane. A Complete list of functional assignment of *MYB* genes is given in Additional file 2: Table S2.

### Gene structure and intron distribution

**Figure 2:**
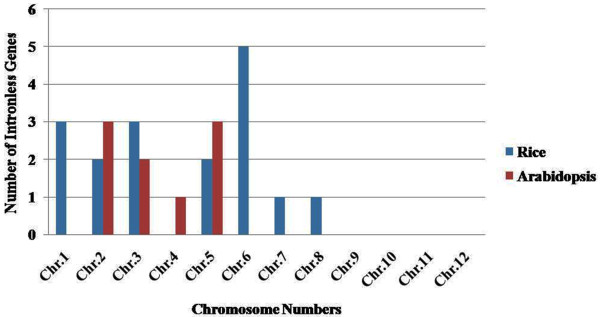
**Chromosome-wise distribution of intronless* MYB* genes in rice and Arabidopsis.**

**Figure 3:**
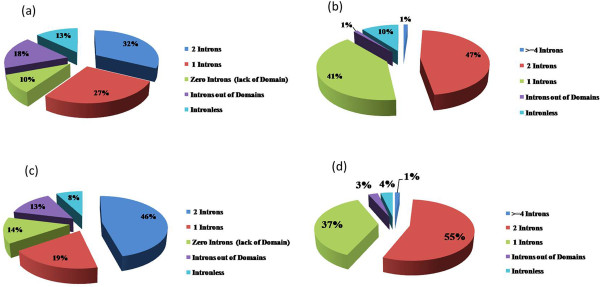
**Intron distribution within the MYB domains of *MYB *genes in rice and Arabidopsis.** The graph shows dominantly two intron positions within the domain of MYB-related (**a**, **c**) and R2R3-MYB genes (**b**, **d**) in rice and Arabidopsis, respectively.

**Figure 4:**
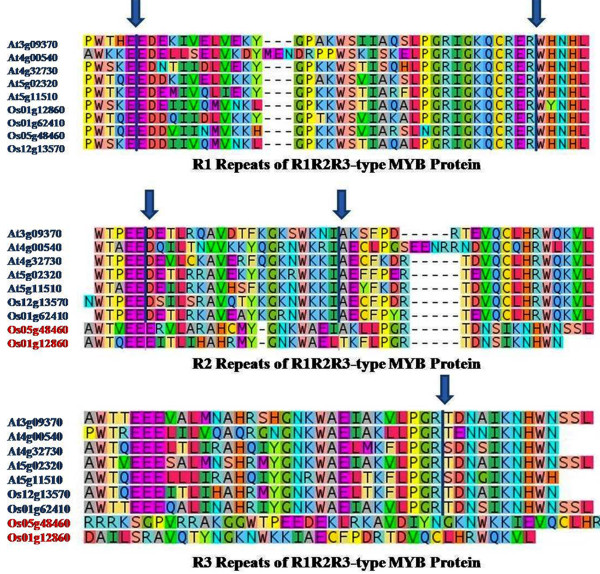
**Conserved intron position within the MYB domain of R1R2R3-type *MYB *genes in rice and Arabidopsis.** Vertical bar and arrow indicate conserved introns position. MSU Gene IDs in red letters represent genes with non-conserved intron position.

To understand the structural components of *MYB* genes, their exon and intron organization was analyzed. We observed that 17 (10.96%) *OsMYB* and 9 (4.56%) *AtMYB* genes were intronless (Figure 2), which is in conformity with the previous analysis [58]. To identify conserved intronless *MYB* genes, blastall (BLASTP) was performed between protein sequence of all the predicted intronless genes of rice and Arabidopsis, and *vice versa*. Expected cut-off value of 1e-6 or less was used to identify the conserved intronless genes. We found that 13 (76.47%) and 7 (77.77%) intronless *OsMYB* and *AtMYB* genes, respectively, were orthologs. Other intronless *MYB* genes that fulfilled the matching criteria, expected cut-off value of 1e-10 or less were referred to as paralogs. We observed that 4 (23.52%) and 2 (22.22%) intronless *OsMYB* and *AtMYB* genes, respectively, were paralogs (Additional file 3: Table S3). This analysis showed that intronless genes of rice and Arabidopsis are highly conserved, and may be involved in similar regulatory functions in these plants [36,58]. To explore the intron density in *MYB* genes with introns, we divided ORF into three zones, namely N-terminal, central and C-terminal zones. We observed that mid region had high density of introns, i.e., 43.99 and 50.63% in rice and Arabidopsis, respectively. The number of introns per ORF varied, with maximum of 12 and 15 introns in *OsMYB4R1* and AT2G47210, respectively. Rice LOC_Os01g43180 and Arabidopsis AT3G10585 genes contain shortest introns with 37 and 43nt, respectively. Among all *MYB* genes, LOC_Os08g25799 of rice and AT1G35515 of Arabidopsis contained longest intron with an intron length of 5116 and 1621nt, respectively (Additional file 4: Table S4). In order to gain insight into exon-intron architecture, the intron positions on MYB domains were investigated. In support with previous results [16,59], we also noticed that a large number of rice (26.45%) and Arabidopsis (38.57%) R2R3-type domain containing proteins have a conserved splicing pattern with three exons and two introns. However, some R2R3-type MYB genes lack one intron either in R2 or R3 repeat in rice (23.22%) and Arabidopsis (25.88%) (Figure 3). It has been proposed that the duplication of R2 in an early form of two repeat MYB proteins gave rise to the R1R2R3 MYB domains [17]. Hence, we also investigated the exon-intron structure of R1R2R3-type MYB proteins. We observed that 3R-MYB proteins contained conserved three exons-two introns pattern in R1 and R2 and one conserved intron in R3 repeat in Arabidopsis. Similarly, in rice, three out of five 3R-MYB genes have similar structure (Figure 4; Additional file 4: Table S4). These results indicate similar distribution of introns in MYB domain in both rice and Arabidopsis.

### Chromosomal distribution, tandem repeats and duplication

**Figure 5:**
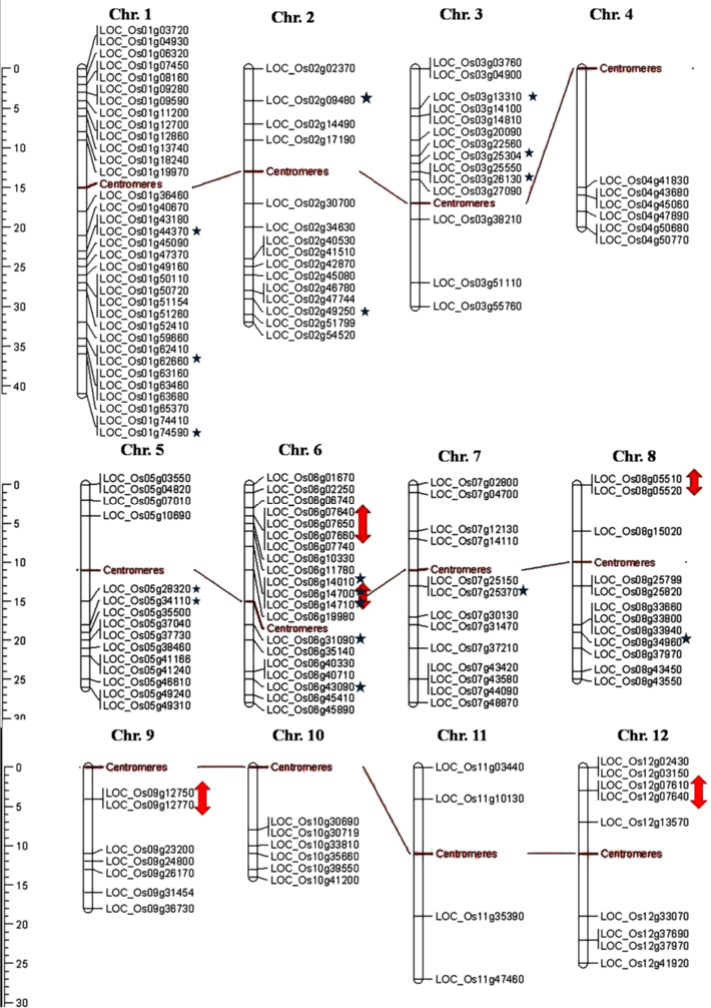
**Distribution of *OsMYB *genes in rice genome.** Arrow and star signs represent to tandem repeats and intronless genes, respectively.

**Figure 6:**
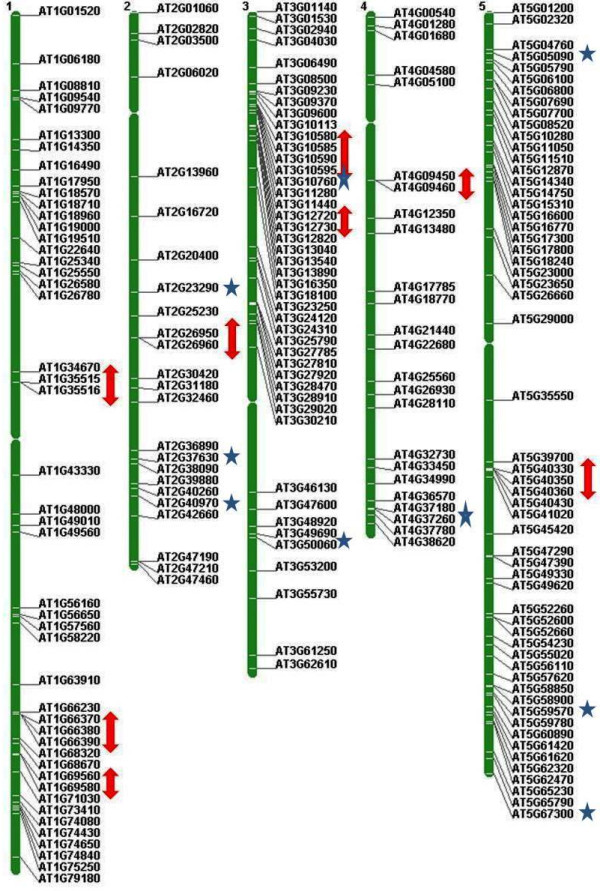
**Distribution of *AtMYB *genes in Arabidopsis genome.** Arrow and star signs represent tandem repeats and intronless genes, respectively.

The position of all 155 *OsMYB* and 197 *AtMYB* genes were mapped on chromosome pseudomolecules available at MSU (release 5) for rice and TAIR (release 8) for Arabidopsis (Figures 5 and 6). The distribution and density of the *MYB* genes on chromosomes were not uniform. Some chromosomes and chromosomal regions have high density of the *MYB* genes than other regions. Rice chromosome 1 and Arabidopsis chromosome 5 contained highest density of *MYB* genes, i.e. 21.93 and 28.93%, respectively. Conversely, chromosome 11 of rice and chromosome 2 of Arabidopsis contained lowest density of *MYB* genes, i.e. 2.58 and 12.69%, respectively. Distribution of *MYB* genes on chromosomes revealed that lower arm of chromosomes are rich in *MYB* genes, i.e. 65.16% in rice and 52.79% in Arabidopsis. Distribution pattern also revealed that chromosome 5 in rice, and chromosome 2 and 5 in Arabidopsis contained higher number of *MYB* genes with introns, i.e. 29.41 and 33.33%, respectively. Intronless *MYB* genes are absent in chromosome 4, 9, 10, 11 and 12 in rice, and chromosome 1 in Arabidopsis (Figure 2). Distribution of *MYB* genes on chromosomal loci revealed that 11 (7.09%) in rice and 20 (10.15%) genes in Arabidopsis were found in tandem repeats suggesting local duplication (Table 2). Chromosome 6 in rice and chromosome 1 in Arabidopsis contained higher number of tandem repeats, i.e. 7 genes and showed over-representation of *MYB* genes. Three direct tandem repeats were found on chromosome 6 (LOC_Os06g07640; LOC_Os06g07650; LOC_Os06g07660) in rice, and chromosome 1 (AT1G66370, AT1G66380; AT1G66390) as well as chromosome 5 (AT5G40330; AT5G40350; AT5G40360) in Arabidopsis. Four direct tandem repeats were also observed on chromosome 3 (AT3G10580, AT3G10585, AT3G10590 and AT3G10595) in Arabidopsis. Manual inspection unraveled 44 (28.38 %) and 69 (35.02%) homologous pairs of *MYB* genes in rice and Arabidopsis, respectively evolved due to segmental duplication. We also observed that two homologous pairs in Arabidopsis contained one *MYB* gene and other than that was not classified as *MYB* gene in TAIR (release 10) databases (Table 3). About 44 (28.39%) *OsMYB* and 69 (35.02%) *AtMYB* genes showed homology with multiple genes including *MYB* genes from various locations on different chromosomes. It is widely accepted that redundant duplicated genes will be lost from the genome due to random mutation and loss of function, except when neo-or sub-functionalization occur [60,61]. Rabinowicz et al. (1999) suggested that gene duplications in R2R3-type *MYB* family occurred during earlier period of evolution in land plants [62]. Recently, a range of duplicated pair of *MYB* genes in R2R3-type protein family has been identified in maize [63]. Among the tandem repeat pair (AT2G26950 and AT2G26960) in Arabidopsis, *AtMYB104* (AT2G26950) is down-regulated by ABA, anoxia and cold stress, but up-regulated under drought, high temperature and salt, while *AtMYB81* (AT2G26960) expression pattern was opposite to that of *AtMYB104*, i.e., *AtMYB81* is up-regulated in response to ABA, anoxia and cold stress, but down regulated under drought, high temperature and salt stresses. Similar diversification was also observed in the duplicate pair (LOC_Os10g33810 and LOC_Os02g41510) in rice. *OsMYB15* (LOC_Os10g33810) expressed in leaf, while LOC_Os02g41510 expressed in shoot and panicle tissue. These spatial and temporal differences among different *MYB* genes evolved by duplication indicate their functional diversification.

**Table 2:** **Comparison of tandem repeat *MYB *genes in rice and Arabidopsis based on cellular localization**

	**Tandem repeat in rice**						**Blast 2 sequences alignment**		
**TR**_**NO**	**TR**_**OsMYB**_**G1**	**TR**_**OsMYB**_**G2**	**OsMYB**_**G1**	**OsMYB**_**G2**	**Cellular localization G1**	**Cellular localization G2**	**Bit score**	**%identity**	**E**-**value**
OsTR1	LOC_Os06g07640	LOC_Os06g07650	OsMYB	OsMYB	Nuclear	Nuclear	75.5	55%	2.00E-18
	LOC_Os06g07650	LOC_Os06g07660	OsMYB	OsMYB	Nuclear	Nuclear	488	84%	2.00E-142
OsTR2	LOC_Os06g14700	LOC_Os06g14710	OsMYB	OsMYB	Nuclear	Nuclear	146	64%	2.00E-40
OsTR3	LOC_Os08g05510	LOC_Os08g05520	OsMYB	OsMYB103	Nuclear	Nuclear	19.2	25%	1.60E-01
OsTR4	LOC_Os09g12750	LOC_Os09g12770	OsMYB	OsMYB	Nuclear	Nuclear	55.8	40%	6.00E-13
OsTR5	LOC_Os12g07610	LOC_Os12g07640	OsMYB	OsMYB	Nuclear	Nuclear	105	45%	2.00E-27
	**Tandem repeat in Arabidopsis**						**Blast 2 sequences alignment**		
**TR**_**NO**	**TR**_**AtMYB**_**G1**	**TR**_**AtMYB**_**G2**	**AtMYB**_**G1**	**AtMYB**_**G2**	**Cellular localization G 1**	**Cellular localization G2**	**Bit score**	**%identity**	**E**-**value**
AtTR1	AT1G35515	AT1G35516	AtMYB8	AtMYB	Nuclear	Nuclear	No significant similarity found		
AtTR2	AT1G66370	AT1G66380	AtMYB113	AtMYB114	Nuclear	Nuclear	212	80%	3.00E-60
	AT1G66380	AT1G66390	AtMYB114	AtMYB90	Nuclear	Nuclear	220	87%	1.00E-62
AtTR3	AT1G69560	AT1G69580	AtMYB105	AtMYB	Nuclear	Nuclear	14.2	31%	5.3
AtTR4	AT2G26950	AT2G26960	AtMYB104	AtMYB81	Nuclear	Nuclear	358	50%	2.00E-103
AtTR5	AT3G10580	AT3G10585	AtMYB	AtMYB	Nuclear	Nuclear	172	64%	4.00E-48
	AT3G10590	AT3G10595	AtMYB	AtMYB	Nuclear	Nuclear	56.6	27%	3.00E-13
AtTR6	AT3G12720	AT3G12730	AtMYB67	AtMYB	Nuclear	Nuclear	16.9	31%	4.40E-01
AtTR7	AT4G09450	AT4G09460	AtMYB	AtMYB6	Cytoplasmic	Nuclear	21.2	25%	1.40E-02
AtTR8	AT5G40330	AT5G40350	AtMYB23	AtMYB24	Nuclear	Nuclear	142	55%	5.00E-39
	AT5G40350	AT5G40360	AtMYB24	AtMYB115	Nuclear	Nuclear	89.4	42%	8.00E-23

*MYB* coding sequence were aligned using BLAST 2 SEQUENCES to quantitate the sequence differences between the paired genes.

**Table 3:** **Comparison of homologous pair of *MYB *genes of rice and Arabidopsis based on cellular localization**

	**Duplications in rice**						**Blast 2 sequences alignment**		
**HP**_**NO**	**OsMYB**_**HP**_**G1**	**OsMYB**_**HP**_**G2**	**OsMYB**_**G1**	**OsMYB**_**G2**	**Cellular localization G 1**	**Cellular localization G2**	**Bit score**	**%identity**	**E**-**value**
OsHP1	LOC_Os01g06320	LOC_Os05g07010	OsMYB	OsMYB	Nuclear	Nuclear	160	81%	1.00E-38
OsHP2	LOC_Os01g18240	LOC_Os05g04820	OsMYB	OsMYB	Nuclear	Nuclear	1230	79%	0.00E+00
OsHP3	LOC_Os01g44370	LOC_Os05g50350	OsMYB	OsMYB	Nuclear	Nuclear	234	82%	8.00E-59
OsHP4	LOC_Os01g47370	LOC_Os05g49240	OsMYB	OsMYB	Nuclear	Nuclear	188	77%	3.00E-47
OsHP5	LOC_Os01g49160	LOC_Os05g48010	OsMYB	OsMYB	Nuclear	Nuclear	234	94%	2.00E-58
OsHP6	LOC_Os01g50720	LOC_Os05g46610	OsMYB	OsMYB	Nuclear	Nuclear	696	77%	0.00E+00
OsHP7	LOC_Os01g59660	LOC_Os05g41166	GAMYB	OsMYB	Nuclear	Nuclear	298	78%	1.00E-75
OsHP8	LOC_Os01g62410	LOC_Os05g38460	OsMYB3R-2	OsMYB	Nuclear	Nuclear	476	74%	8.00E-124
OsHP9	LOC_Os01g63460	LOC_Os05g37730	OsMYB	OsMYB	Nuclear	Nuclear	22	100%	6.80E-01
OsHP10	LOC_Os01g65370	LOC_Os05g35500	OsMYB3	OsMYB	Nuclear	Nuclear	636	88%	6.00E-168
OsHP11	LOC_Os02g09480	LOC_Os05g37730	OsMYB	OsMYB	Nuclear	Nuclear	32	87%	7.00E-04
OsHP12	LOC_Os02g14490	LOC_Os06g35140	OsMYB	OsMYB	Nuclear	Nuclear	548	73%	2.00E-143
OsHP13	LOC_Os02g40530	LOC_Os04g42950	OsMYB	OsMYB	Nuclear	Nuclear	284	94%	8.00E-72
OsHP14	LOC_Os02g41510	LOC_Os04g43680	OsMYB	OsMYB4	Nuclear	Nuclear	460	86%	3.00E-120
OsHP15	LOC_Os02g42870	LOC_Os04g45060	OsMYB	OsMYB	Nuclear	Nuclear	744	77%	0.00E+00
OsHP16	LOC_Os02g45080	LOC_Os04g47890	OsMYB	OsMYB	Nuclear	Nuclear	312	73%	6.00E-80
OsHP17	LOC_Os02g46780	LOC_Os04g50770	OsMYB	OsMYB	Nuclear	Nuclear	620	70%	2.00E-163
OsHP18	LOC_Os02g51799	LOC_Os06g11780	OsMYB	OsMYB93	Nuclear	Nuclear	442	80%	5.00E-115
OsHP19	LOC_Os02g54520	LOC_Os07g48870	OsMYB	OsMYB2	Nuclear	Nuclear	54	78%	1.00E-09
OsHP20	LOC_Os03g03760	LOC_Os10g39550	OsMYB	OsMYB	Nuclear	Nuclear	136	83%	3.00E-31
OsHP21	LOC_Os03g20090	LOC_Os07g48870	OsMYB112	OsMYB2	Nuclear	Nuclear	554	84%	2.00E-145
OsHP22	LOC_Os03g25550	LOC_Os07g44090	OsMYB	OsMYB	Nuclear	Nuclear	374	88%	1.00E-96
OsHP23	LOC_Os03g26130	LOC_Os07g43580	OsMYB	OsMYB30	Nuclear	Nuclear	384	82%	2.00E-99
OsHP24	LOC_Os05g04820	LOC_Os07g44090	OsMYB	OsMYB	Nuclear	Nuclear	422	83%	2.00E-109
OsHP25	LOC_Os05g10690	LOC_Os01g09640	OsMYB	OsMYB	Nuclear	Nuclear	232	83%	9.00E-58
OsHP26	LOC_Os05g49240	LOC_Os05g50340	OsMYB	OsMYB	Nuclear	Nuclear	104	72%	4.00E-24
OsHP27	LOC_Os06g43090	LOC_Os02g09480	OsMYB	OsMYB	Nuclear	Nuclear	616	71%	2.00E-162
OsHP28	LOC_Os06g45410	LOC_Os02g07770	OsMYB	OsMYB	Nuclear	Nuclear	180	90%	1.00E-43
OsHP29	LOC_Os06g45890	LOC_Os02g07170	OsMYB	OsMYB	Nuclear	Nuclear	98	81%	1.00E-21
OsHP30	LOC_Os07g02800	LOC_Os03g55590	OsMYB	OsMYB	Nuclear	Nuclear	162	91%	1.00E-38
OsHP31	LOC_Os08g25799	LOC_Os09g12750	OsMYB	OsMYB	Nuclear	Nuclear	682	80%	2.00E-180
OsHP32	LOC_Os08g25820	LOC_Os09g12770	OsMYB	OsMYB	Nuclear	Nuclear	616	73%	2.00E-162
OsHP33	LOC_Os08g33660	LOC_Os02g36890	OsMYB16	OsMYB	Nuclear	Nuclear	134	69%	4.00E-31
OsHP34	LOC_Os08g33660	LOC_Os04g38740	OsMYB16	OsMYB	Nuclear	Nuclear	136	80%	1.00E-31
OsHP35	LOC_Os08g33940	LOC_Os09g24800	OsMYB	OsMYB	Nuclear	Nuclear	838	76%	0.00E+00
OsHP36	LOC_Os08g43450	LOC_Os09g36250	OsMYB	OsMYB	Nuclear	Nuclear	76	71%	2.00E-15
OsHP37	LOC_Os08g43550	LOC_Os09g36730	OsMYB7	OsMYB	Nuclear	Nuclear	502	84%	1.00E-131
OsHP38	LOC_Os09g23200	LOC_Os08g33050	OsMYB	OsMYB	Nuclear	Nuclear	222	66%	2.00E-54
OsHP39	LOC_Os10g33810	LOC_Os02g41510	OsMYB15	OsMYB	Nuclear	Nuclear	374	81%	8.00E-97
OsHP40	LOC_Os10g33810	LOC_Os04g43680	OsMYB15	OsMYB4	Nuclear	Nuclear	384	82%	2.00E-99
OsHP41	LOC_Os10g39550	LOC_Os03g03760	OsMYB	OsMYB	Nuclear	Nuclear	384	81%	3.00E-99
OsHP42	LOC_Os11g03440	LOC_Os12g03150	OsMYB	OsMYB	Nuclear	Nuclear	1702	96%	0.00E+00
OsHP43	LOC_Os11g47460	LOC_Os12g37970	OsMYB	OsMYB	Nuclear	Nuclear	634	83%	2.00E-167
OsHP44	LOC_Os12g37690	LOC_Os11g45740	OsMYB78	OsMYB	Nuclear	Nuclear	226	88%	5.00E-56
	**Duplications in*Arabidopsis***						**Blast 2 sequences alignment**		
**HP**_**NO**	**AtMYB**_**HP**_**G1**	**AtMYB**_**HP**_**G2**	**AtMYB**_**G1**	**ATMYB**_**G2**	**Cellular localization G 1**	**Cellular localization G2**	**Bit score**	**%identity**	**E**-**value**
AtHP1	AT2G31180	AT1G06180	AtMYB14	AtMYB13	Nuclear	Nuclear	350	84%	2.00E-100
AtHP2	AT1G57560	AT1G09540	AtMYB50	AtMYB61	Nuclear	Nuclear	392	88%	7.00E-113
AtHP3	AT1G58220	AT1G09710	AtMYB1l	AtMYB	Nuclear	Nuclear	827	75%	0
AtHP4	AT1G26580	AT1G13880	AtMYB	No MYB	Nuclear	Nuclear	45.4	76%	4.00E-08
AtHP5	AT2G02820	AT1G14350	AtMYB88	AtMYB124	Nuclear	Nuclear	728	80%	0
AtHP6	AT3G12820	AT1G16490	AtMYB10	AtMYB58	Nuclear	Nuclear	293	79%	3.00E-83
AtHP7	AT1G17950	AT1G73410	AtMYB52	AtMYB54	Nuclear	Nuclear	381	88%	7.00E-110
AtHP8	AT1G79180	AT1G16490	AtMYB63	AtMYB58	Nuclear	Nuclear	346	84%	4.00E-99
AtHP9	AT5G61420	AT1G18570	AtMYB28	AtMYB51	Nuclear	Nuclear	99	86%	1.00E-24
AtHP10	AT1G74080	AT1G18570	AtMYB122	AtMYB51	Nuclear	Nuclear	305	81%	9.00E-87
AtHP11	AT5G07700	AT1G18570	AtMYB76	AtMYB51	Nuclear	Nuclear	185	71%	2.00E-50
AtHP12	AT5G60890	AT1G18570	AtMYB34	AtMYB51	Nuclear	Nuclear	206	77%	8.00E-57
AtHP13	AT1G74430	AT1G18710	AtMYB95	AtMYB47	Nuclear	Nuclear	351	82%	7.00E-101
AtHP14	AT1G74840	AT1G19000	AtMYB	AtMYB	Nuclear	Nuclear	233	85%	3.00E-65
AtHP15	AT1G35516	AT1G22640	AtMYB	AtMYB3	Nuclear	Nuclear	No significant similarity found		
AtHP16	AT4G09460	AT1G22640	AtMYB6	AtMYB3	Nuclear	Nuclear	394	84%	1.00E-113
AtHP17	AT1G68320	AT1G25340	AtMYB62	AtMYB116	Nuclear	Nuclear	366	86%	3.00E-105
AtHP18	AT3G27810	AT1G25340	AtMYB21	AtMYB116	Nuclear	Nuclear	149	70%	7.00E-40
AtHP19	AT1G68670	AT1G25550	AtMYB	AtMYB	Nuclear	Nuclear	176	84%	8.00E-48
AtHP20	AT3G29020	AT1G26780	AtMYB110	AtMYB117	Nuclear	Nuclear	232	77%	8.00E-65
AtHP21	AT1G26780	AT1G69560	AtMYB117	AtMYB105	Nuclear	Nuclear	416	88%	3.00E-120
AtHP22	AT5G39700	AT1G69560	AtMYB89	AtMYB105	Nuclear	Nuclear	No significant similarity found		
AtHP23	AT5G07690	AT1G74080	AtMYB29	AtMYB122	Nuclear	Nuclear	161	76%	2.00E-43
AtHP24	AT1G19510	AT1G75250	AtMYB	AtMYB	Nuclear	Nuclear	154	80%	4.00E-42
AtHP25	AT4G36570	AT1G75250	AtMYB	AtMYB	Nuclear	Nuclear	No significant similarity found		
AtHP26	AT4G34990	AT2G16720	AtMYB32	AtMYB7	Nuclear	Nuclear	411	85%	1.00E-118
AtHP27	AT4G37260	AT2G23290	AtMYB73	AtMYB70	Nuclear	Nuclear	364	84%	1.00E-104
AtHP28	AT5G67300	AT2G23290	AtMYB44	AtMYB70	Nuclear	Nuclear	171	77%	3.00E-46
AtHP29	AT5G11050	AT2G25230	AtMYB64	AtMYB100	Nuclear	Nuclear	63.9	78%	1.00E-13
AtHP30	AT5G01200	AT2G38090	AtMYB	AtMYB	Nuclear	Nuclear	195	82%	1.00E-53
AtHP31	AT3G55730	AT2G39880	AtMYB109	AtMYB25	Nuclear	Nuclear	281	81%	2.00E-79
AtHP32	AT3G10760	AT2G40970	AtMYB	AtMYB	Nuclear	Nuclear	235	69%	8.00E-66
AtHP33	AT5G05090	AT2G40970	AtMYB	AtMYB	Nuclear	Nuclear	156	81%	5.00E-42
AtHP34	AT3G62610	AT2G47460	AtMYB11	AtMYB12	Nuclear	Nuclear	388	86%	9.00E-112
AtHP35	AT5G15310	AT3G01140	AtMYB16	AtMYB106	Nuclear	Nuclear	593	83%	2.00E-173
AtHP36	AT5G40350	AT3G01530	AtMYB24	AtMYB57	Nuclear	Nuclear	254	81%	1.00E-71
AtHP37	AT5G16600	AT3G02940	AtMYB43	AtMYB107	Nuclear	Nuclear	110	73%	7.00E-28
AtHP38	AT5G16770	AT3G02940	AtMYB9	AtMYB107	Nuclear	Nuclear	586	86%	3.00E-171
AtHP39	AT3G24120	AT3G04030	AtMYB3l	AtMYB	Nuclear	Nuclear	73%	86	1.00E-20
AtHP40	AT5G18240	AT3G04030	AtMYB	AtMYB	Nuclear	Nuclear	887	80%	0
AtHP41	AT5G49620	AT3G06490	AtMYB78	AtMYB108	Nuclear	Nuclear	396	83%	4.00E-114
AtHP42	AT5G02320	AT3G09370	AtMYB3R5	AtMYB3R3	Nuclear	Nuclear	610	85%	4.00E-178
AtHP43	AT5G04760	AT3G10580	AtMYB	AtMYB	Nuclear	Nuclear	105	71%	7.00E-27
AtHP44	AT5G05790	AT3G11280	AtMYB	AtMYB	Nuclear	Nuclear	455	80%	5.00E-132
AtHP45	AT5G06100	AT3G11440	AtMYB33	AtMYB65	Nuclear	Nuclear	710	78%	0
AtHP46	AT1G56160	AT3G12820	AtMYB72	AtMYB10	Nuclear	Nuclear	270	81%	2.00E-76
AtHP47	AT4G13480	AT3G24310	AtMYB79	AtMYB71	Nuclear	Nuclear	436	83%	2.00E-126
AtHP48	AT1G13300	AT3G25790	AtMYB	AtMYB	Nuclear	Nuclear	250	84%	4.00E-70
AtHP49	AT5G40360	AT3G27785	AtMYB115	AtMYB118	Nuclear	Nuclear	161	76%	3.00E-43
AtHP50	AT3G01530	At1g68320	AtMYB57	AtMYB62	Nuclear	Nuclear	239	81%	4.00E-67
AtHP51	AT5G14750	AT3G27920	AtMYB66	AtMYB0	Nuclear	Nuclear	320	80%	1.00E-91
AtHP52	AT5G40330	AT3G27920	AtMYB23	AtMYB0	Nuclear	Nuclear	379	85%	2.00E-109
AtHP53	AT5G59780	AT3G46130	AtMYB59	AtMYB48	Nuclear	Nuclear	237	86%	1.00E-66
AtHP54	AT5G59570	AT3G46640	AtMYB	AtMYB	Nuclear	Nuclear	313	85%	4.00E-89
AtHP55	AT5G62470	AT3G47600	AtMYB96	AtMYB94	Nuclear	Nuclear	527	88%	2.00E-153
AtHP56	AT5G65790	AT3G49690	AtMYB68	AtMYB84	Nuclear	Nuclear	494	87%	2.00E-143
AtHP57	AT4G37780	AT3G49690	AtMYB87	AtMYB84	Nuclear	Nuclear	246	79%	4.00E-69
AtHP58	AT4G22680	AT3G61250	AtMYB85	AtMYB17	Nuclear	Nuclear	147	70%	3.00E-39
AtHP59	AT1G01520	AT4G01280	AtMYB	AtMYB	Nuclear	Nuclear	272	83%	7.00E-77
AtHP60	AT4G21440	AT4G05100	AtMYB102	AtMYB74	Nuclear	Nuclear	385	89%	1.00E-110
AtHP61	AT5G52260	AT4G25560	AtMYB19	AtMYB18	Nuclear	Nuclear	407	79%	2.00E-117
AtHP62	AT5G55020	AT4G26930	AtMYB120	AtMYB97	Nuclear	Nuclear	283	82%	7.00E-80
AtHP63	AT2G20400	AT4G28610	AtMYB	No MYB	Nuclear	Nuclear	419	73%	7.00E-121
AtHP64	AT5G11510	AT4G32730	AtMYB3R4	AtMYB3R1	Nuclear	Nuclear	329	78%	3.00E-93
AtHP65	AT3G09600	AT5G02840	AtMYB	MYB (LCL1)	Nuclear	Nuclear	682	80%	0
AtHP66	AT3G10590	AT5G04760	AtMYB	AtMYB	Nuclear	Nuclear	51.8	76%	1.00E-10
AtHP67	AT5G23650	AT5G08520	AtMYB	AtMYB	Nuclear	Nuclear	139	72%	8.00E-37
AtHP68	AT5G65230	AT5G10280	AtMYB53	AtMYB92	Nuclear	Nuclear	534	84%	9.00E-156
AtHP69	AT3G50060	AT5G67300	AtMYB77	AtMYB44	Nuclear	Nuclear	265	82%	1.00E-74

The coding sequence were aligned using BLAST 2 SEQUENCES to quantitate the sequence differences between the gene pairs.

### *Cis*-motifs in the MYB gene promoters

**Figure 7:**
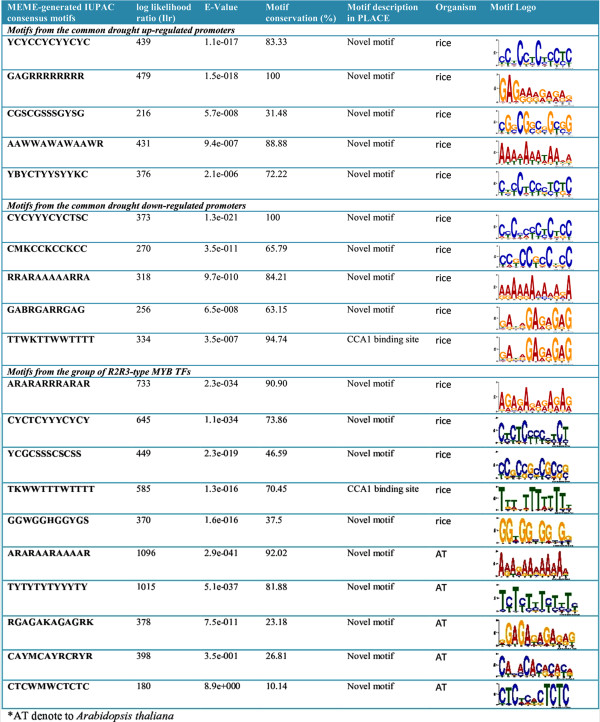
**Conserved *cis*-motifs found in upstream promoter region of *MYB *genes in rice and Arabidopsis. a**) Motifs from the promoter region of drought stress-regulated *MYB* genes in rice, **b**) Motifs from the group of R2R3-*MYB* genes in both rice and Arabidopsis.

Discovery of regulatory *cis*-elements in the promoter regions is essential to understand the spatial and temporal expression pattern of *MYB* genes. Co-expressed genes may be regulated by a common set of transcription factors, and can be detected by the occurrence of specific *cis*-regulatory motifs in the promoter region. Hence, we analyzed the promoter regions of the drought up- and down-regulated *MYB* genes identified from our previous microarray data experiments [64]. Among the top five *cis*-motifs identified by this analysis, only CCA1 (TTWKTTWWTTTT) was the previously known *cis*-motif. Although, CCA1 *cis*-motif was reported as common feature of rice genome [65], we found CCA1 *cis*-motif only in genes that are down-regulated by drought stress (Figure 7). The CCA1 motif was found in 94.74% of the drought down-regulated genes in rice. Furthermore, we investigated the group of R2R3-type *MYB* genes for the discovery of gene-specific new *cis*-regulatory element in both rice and Arabidopsis. Likewise, we discovered novel *cis*-motifs with no description in PLACE database, except for CCA1 motif in rice (Figure 7). The CCA1 motif was found in 70.45% of the R2R3-type *MYB* genes in rice. The CCA1, a MYB-related TF, binds to CCA1 motif and regulate circadian clock controlled expression of genes in Arabidopsis [66]. To validate our prediction, we examined the diurnal or circadian clock controlled *MYB* expression using “Diurnal Version 2.0” [67]. About 47.74 and 90.86% *MYB* genes were found to be diurnal/circadian-regulated in rice and Arabidopsis, respectively (Additional file 5: Table S5). Noticeably, we did not find any common motif between rice and Arabidopsis *MYB* promoter regions, indicating divergence in regulatory region of *MYB* genes between monocot and dicot species.

### Expression of *MYB* genes under abiotic stresses

To identify *MYB* genes with a potential role in abiotic stress response of plants, we analyzed the expression pattern of *MYB* genes in response to abiotic stresses. Expression of *MYBs* genes was examined from the availability of full-length cDNA (FL-cDNA) and Expressed Sequence Tag (EST) available at MSU and dbEST databases for rice and Arabidopsis, respectively [68]. It was found that 109 *OsMYB* genes in rice and 157 *AtMYB* genes in Arabidopsis had one or more representative ESTs. The LOC_Os10g41200 and AT5G47390 gene in rice and Arabidopsis had maximum number of ESTs, that is, 219 and 44, respectively. About 70% of rice *MYB* genes and 80% of Arabidopsis *MYB* genes appeared to be highly expressed as evident from the availability of ESTs for these genes (Additional file 6: Table S6). Further, we assessed the expression levels of *MYB* genes under various abiotic stresses by PlantQTL-GE [69], GENEVESTIGATOR [70,71] and our previous microarray data experiment (E-MEXP-2401) with rice cv. Nagina 22 and IR64 under normal and drought conditions (Additional file 7: Table S7). In our previous microarray data experiments, we found that 142 (92.26%) *MYB* genes were expressed in seedlings of rice (Additional file 8: Figure S1), of which 92 genes were differentially regulated under drought stress. In IR64, 30 genes were up-regulated (≥ 2.0 fold) and 30 genes were down-regulated (≤ 2.0 fold), while in Nagina 22, 22 genes were up-regulated (≥ 2.0 fold) and 19 genes were down-regulated (≤ 2.0 fold) under drought stress. The exploration of PlantQTL-GE for rice *MYBs* showed that 14 (9.03%) *OsMYB* genes were up-regulated under cold, drought and salt stress in rice, of which 10 are up-regulated under drought condition. These results suggest that large set of *MYB* genes may have a role in drought stress response in rice. Previous studies have shown that over-expression of *MYB* genes improved abiotic stress tolerance of rice and Arabidopsis [24,72]. In addition to these, we have identified additional *MYB* genes that are regulated by drought and other stresses, and thus can be used as candidate genes for functional validation. The GENEVESTIGATOR analysis showed that 44.67, 41.12 and 56.85% *AtMYB* genes were down regulated and 47.21, 50.76 and 35.02% *AtMYB* genes were up regulated in cold, drought and salt stress, respectively (Additional file 9: Figure S2a, b and c, Additional file 10: Figure S3).

**Figure 8:**
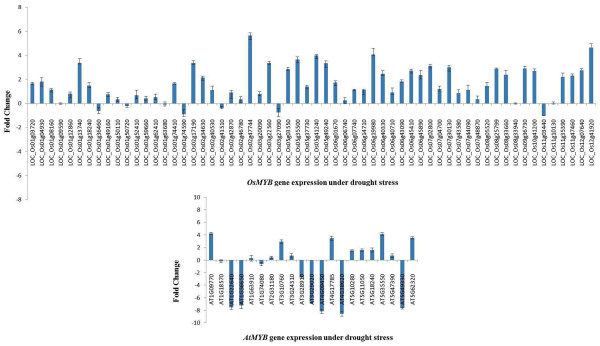
**QRT-PCR expression analyses of*OsMYB*and*AtMYB*genes under drought stress in rice and Arabidopsis.**

We analyzed expression patterns of 60 *OsMYB* and 21 *AtMYB* genes using QRT-PCR. These genes were selected based on phylogenetic analysis and one gene from each cluster was selected for expression analysis. Out of the 60 genes examined by QRT-PCR, 28 *OsMYB* genes were up-regulated (≥ 1.5 fold change) under drought stress in rice cv. Nagina 22 (Figure 8). We also found that LOC_Os02g47744, LOC_Os12g41920 and LOC_Os06g19980 were highly up-regulated (≥ 4 fold change), indicating their potential role in drought stress. QRT-PCR analysis of 21 *MYB* genes in Arabidopsis revealed that 7 *AtMYB* genes were up-regulated (≥ 1.5 fold changes) and another 7 *AtMYB* genes were down-regulated (≤ 1.5 fold change) under drought stress (Figure 8).

### Tissue-specific expression

In rice, a tissue breakdown of EST evidence for *MYB* genes was analyzed using the Rice Gene Expression Anatomy Viewer, MSU database [73,74]. In case of Arabidopsis, tissue-specific expressions of *MYB* genes were obtained from GENEVESTIGATOR tool [70,71]. The expression patterns of *MYB* genes in different tissues are listed in Additional file 11: Table S8. The results showed that large numbers of *OsMYB* genes (32.90%) were highly expressed in the panicle, leaf and shoots (Additional file 12: Figure S4). EST frequency analysis suggested that *OsMYB g*enes, LOC_Os02g34630, LOC_Os08g05510, LOC_Os01g74590, LOC_Os02g09480, LOC_Os09g36730, *OsMYB4*, LOC_Os10g41200 and LOC_Os01g13740 are highly expressed in flower, anther, endosperm, pistil, shoot, panicle, immature seed and whole plant, respectively. In case of leaves, we observed that three *MYB* genes, i.e., *OsMYB48*, LOC_Os06g40710 and LOC_Os10g41200 showed highest levels of expression. In Arabidopsis, the following *MYB* genes expressed at a very high level: *AtMYBCDC5* in callus and seed; AT1G19000 in seedling and stem; AT1G74840 in root and root tip; AT1G26580 in flower, *AtMYB91* in shoot, and *AtMYB44* in pedicel and leaves. In wheat, *TaMYB1* showed high expression in root, sheath and leaf, while *TaMYB2* expression was highest in root and leaf, but at low in sheath [75]. *TaMYB1* and *TaMYB2* showed a very high sequence similarity with *AtMYB44* and *OsMYB48*, respectively. Our analysis also revealed that these two *MYBs* are highly expressed in leaf as in case of wheat. These analyses will be useful in selecting candidate genes for functional analysis of their role in a specific tissue.

### Evolutionary relationship

To understand the evolutionary relationship among *MYB* family genes, phylogenetic trees were constructed using the multiple sequence alignment of MYB proteins [76]. The tree revealed that tandem repeat and homologous pairs were grouped together into single clade with very strong bootstrap support (Additional file 13: Figure S5). These results further support gene duplication in rice and Arabidopsis during evolution which may allow functional diversification by adaptive protein structures [77]. It was also noticed that few “homologues pairs” (e.g. AT5G16600-AT3G02940 in Arabidopsis; LOC_Os12g07610- LOC_Os12g07640 in rice) and “tandem repeat pairs” (e.g. AT3G12720-AT3G12730 in Arabidopsis; LOC_Os06g14700-LOC_Os06g14710 in rice) were found in distinct clade, indicating that only few members had common ancestral origin that existed before the divergence of monocot and dicot. MYB proteins from rice and Arabidopsis with same number of MYB domains were grouped into a single clade. For instance, all the MYBs belonging to R1R2R3 family in both rice and Arabidopsis were clustered into single clade. Within the R2R3 clade, *MYBs* from rice and Arabidopsis were not found in distinct groups. These results suggest that significant expansion of R2R3-type *MYB* genes in plants occurred before the divergence of monocots and dicots, which in agreement with the previous studies [4,62]. Finally, we observed that two CDC5-type and one 4-repeat MYB orthologs were clustered into single clade and might have been derived from an ancient paralog of widely distributed R2R3 *MYB* genes.

## Conclusions

Our study provides genome-wide comparative analysis of *MYB* TF family gene organization, sequence diversity and expression pattern in rice and Arabidopsis. Structural analysis revealed that introns are highly conserved in the central region of the gene, and R2R3-type MYB proteins usually have two introns at conserved positions. Analysis of length and splicing of the intron/exon and their position in MYB domain suggested that introns were highly conserved within the same subfamily. Most of the *MYB* genes are present as duplicate genes in both rice and Arabidopsis. Phylogenetic analysis of rice and Arabidopsis MYB proteins showed that tandem repeat and homologous pair was grouped together into single clade. Consensus motif analysis of 1kb upstream region of *MYB* gene ORFs led to the identification of conserved and over-represented *cis*-motifs in both rice and Arabidopsis. The comparative analysis of *MYB* genes in rice and Arabidopsis elucidated chromosomal location, gene structure and phylogenetic relationships, and expression analysis led to the identification of abiotic stress responsive and tissue-specific expression pattern of the selected *MYB* genes, suggesting functional diversification. Our comprehensive analyses will help design experiments for functional validation of their precise role in plant development and stress responses.

## Methods

### Identification of *MYB* gene family in rice and Arabidopsis

To identify *MYB* transcription factor family genes, we searched and obtained genes annotated as *MYB* in MSU (release 5) for rice and TAIR (release 8) for Arabidopsis by using in-house PERL script along with careful manual inspection. The primary search disclosed 161 and 199 members annotated as “MYB” or “MYB-related genes” in MSU and TAIR database, respectively. We observed that some protein members lack MYB-DNA binding domain but still annotated as MYB protein family in MSU and TAIR database. We discarded these proteins based in the annotation in MSU (release 7) for rice and TAIR (release 10). Finally, we obtained 155 and 197 *MYB* genes in rice and Arabidopsis, respectively. The gene identifiers were assigned to each *OsMYB* and *AtMYB* genes to avoid confusion when multiple names are used for same gene. Uncharacterized *MYB* genes are denoted here by their locus id.

### MYB annotation

To identify number of domains present in MYB protein we executed domain search by Conserved Domains Database [78] ( http://www.ncbi.nlm.nih.gov/Structure/cdd/cdd.shtml) and pfam database [79] ( http://pfam.sanger.ac.uk/)with both local and global search strategy and expectation cut off (E value) 1.0 was set as the threshold for significance. Only significant domain found in rice and Arabidopsis MYB protein sequence were considered as a valid domain. To get more information about nature of the MYB protein, grand average of hydropathy (GRAVY), PI and the molecular weight were predicted by ProtParam tool available on Expert Protein Analysis System (ExPASy) proteomics server ( http://www.expasy.ch/tools/protparam.html). The subcellular localization of MYB proteins were predicted by Protein Localization Server (PLOC) ( http://www.genome.jp/SIT/plocdir/), Subcellular Localization Prediction of Eukaryotic Proteins (SubLoc V 1.0) ( http://www.bioinfo.tsinghua.edu.cn/SubLoc/eu_predict.htm), SVM based server ESLpred ( http://www.imtech.res.in/raghava/eslpred/submit.html), and ProtComp 9.0 server ( http://linux1.softberry.com/berry.phtml?topic=protcomppl&group=programs&subgroup=proloc). Further, species-specific localization prediction system was utilized for Arabidopsis (AtSubP, http://bioinfo3.noble.org/AtSubP/) [57]. MYB protein function in term of their Gene Ontology (GO) was predicted by GO annotation search page available at MSU ( http://rice.plantbiology.msu.edu/downloads_gad.shtml) and TAIR ( http://www.arabidopsis.org/tools/bulk/go/index.jsp) for rice and Arabidopsis, respectively. Localization consensus was predicted based on majority of result. The confidence level was acquired by assigning equal numeric value (e.g. one) to each general localization predictor and higher value to gene ontology (e.g. two) and species specific predictor (e.g. three).

### Identification of over-represented motifs

We discovered over represented *cis*-motif consensus pattern in 1 kb upstream sequence from translational initiation codon of *MYB* genes in both rice and Arabidopsis using the Multiple Expectation maximization for Motif Elicitation analysis tool [80] (MEME version 4.1.0, http://meme.sdsc.edu/meme/meme-intro.html). This program was used to search best 5 c*is*-motif consensus patterns of 8–12 bases width, with E-value < 0.01, only on the forward strand of the input sequences. Motifs graph were plotted according to their position within the region using WebLogo tool ( http://weblogo.berkeley.edu/logo.cgi). Discovered motifs were analyzed using PLACE [81] ( http://www.dna.affrc.go.jp/PLACE/). Diurnal and circadian controlled *MYB* expression was explored from “Diurnal Version 2.0” (Mockler lab; http://diurnal.mocklerlab.org/).

### Phylogenetic analysis

To generate the phylogenetic trees of *MYB* transcription factor family genes, multiple sequence alignment of MYB protein sequence were performed using COBALT program [82] ( http://www.ncbi.nlm.nih.gov/tools/cobalt/). COBALT program automatically utilize information about *bona fide* proteins (i.e. MYB domains in this case) to execute multiple sequence alignment and build phylogenetic tree. The dendrogram were constructed with the following parameters; method-fast minimum evolution, max sequence difference-0.85, distance- grishin (protein).

### MYB localization, tandem repeat and duplication

To map the gene loci on rice and Arabidopsis chromosomes pseudomolecules were used in MapChart (version 2.2) program [83] for rice and chromosome map tool [84] for Arabidopsis available on The Arabidopsis Information Resource (TAIR) database ( http://www.arabidopsis.org/jsp/ChromosomeMap/tool.jsp). Tandem repeats were identified by manual visualization of rice and Arabidopsis physical map. Duplication or homologous pair genes were obtained by the segmental genome duplication segment ( http://rice.plantbiology.msu.edu/segmental_dup/) and Arabidopsis Syntenic Pairs / Annotation Viewer ( http://synteny.cnr.berkeley.edu/AtCNS/) in rice (distance = 500kb) and Arabidopsis, respectively. The tandem repeat and homologous pairs were aligned with the BLAST 2 SEQUENCE tool available on National Center on Biotechnology Information (NCBI) ( http://blast.ncbi.nlm.nih.gov/Blast.cgi/).

### Gene structure analysis

To know more about intron / exon structure, *MYB* coding sequence (CDS) were aligned with their corresponding genomic sequences using spidey tool available on NCBI ( http://www.ncbi.nlm.nih.gov/spidey/). To identify conserved intronless genes between rice and Arabidopsis, local protein blast (BLASTP) ( http://www.molbiol.ox.ac.uk/analysis_tools/BLAST/BLAST_blastall.shtml) was performed for protein sequences of all predicted intronless genes in rice against all predicted intronless gene in Arabidopsis, and vice versa. Hits with 1e-6 or less were treated as conserved intronless genes and hits with 1e-10 or less were treated as paralogs. The cutoff of sequence identity was considered as ≥ 20% over the 70% average query coverage.

### Expression analysis

Expression support for each gene model is explored through gene expression evidence search page ( http://rice.plantbiology.msu.edu/locus_expression_evidence.shtml) available at MSU for rice and GENEVESTIGATOR tool ( https://www.genevestigator.com/) for Arabidopsis. *MYB* genes for which no ESTs were found, blast (BLASTP and TBLASTN) ( http://blast.ncbi.nlm.nih.gov/Blast.cgi) search using NCBI databases was performed. Significant similarity of *MYB* genes with *MYB* genes of other plant species was searched. To measure the *MYB* expression level in abiotic stress plant QTLGE database was used ( http://www.scbit.org/qtl2gene/new/) for rice and GENEVESTIGATOR tool ( https://www.genevestigator.com/) for Arabidopsis. To identify tissue specific expression level of *OsMYB* genes in rice, highly expressed gene search ( http://Rice.plantbiology.msu.edu/tissue.expression.shtml) available at MSU were used. For Arabidopsis, GENEVESTIGATOR tool ( https://www.genevestigator.com/gv/user/gvLogin.jsp) was used.

### Plant materials and growth conditions

The plant materials used were drought tolerant rice (*Oryza sativa* L. subsp. *Indica*) cv. Nagina 22 and *Arabidopsis thaliana* ecotype Columbia. The seeds were surface sterilized. Rice seeds were placed on absorbent cotton, which was soaked overnight in water and kept in medium size plastic trays. Arabidopsis seeds were germinated on MS-agar medium containing 1% Sucrose and seven days old seedlings were transferred to soilrite for further growth. The rice and Arabidopsis seedlings were grown in a greenhouse under the photoperiod of 16/8 h light/dark cycle at 28°C ± 1 and 23°C ± 1, respectively.

### Drought stress treatment

Drought was imposed to 3-weeks old rice seedlings [85] and 5-week-old Arabidopsis plants by withholding water till visible leaf rolling was observed. Control plants were irrigated with sufficient water. Plant water status was quantified by measuring relative water content of leaf. Control plants showed 96.89 and 97.49% RWC (relative water content), while stressed plants showed 64.86 and 65.2% RWC in rice and Arabidopsis, respectively.

### Real-Time RT-PCR

Total RNA from rice and Arabidopsis were isolated by TRIzol Reagent (Ambion) and treated with DNase (QIAGEN, GmbH). The first strand cDNA of rice and Arabidopsis was synthesized using Superscript III Kit (Invitrogen) from 1 μg of total RNA according to manufacturer’s protocol. Reverse transcription reaction was carried out at 44°C for 60 min followed by 92°C for 10 min. Five ng of cDNA was used as template in a 20 μL RT reaction mixture. Sixty three pairs of rice and 51 pairs of Arabidopsis gene specific primers were used to study expression of *MYB* transcription factor. Gene specific primers were designed using IDT PrimerQuest ( http://www.idtdna.com/scitools/applications/primerquest/default.aspx). Ubiquitin and actin primers were used as an internal control in rice and Arabidopsis, respectively. The primer combinations used here for real-time RT-PCR analysis specifically amplified only one desired band. The dissociation curve testing was carried out for each primer pair showing only one melting temperature. The RT-PCR reactions were carried out at 95°C for 5 min followed by 40 cycles of 95°C for 15s and 60°C for 30s each by the method described previously by Dai et al., 2007 [24]. For qRT-PCR, QuantiFast SYBR Green PCR master mix (QIAGEN GmbH) was used according to manufacturer’s instruction. The threshold cycles (C_T_) of each test target were averaged for triplicate reactions, and the values were normalized according to the C_T_ of the control products (Os-actin or Ubiquitin) in case of rice and Arabidopsis, respectively. *MYB* TFs expression data were normalized by subtracting the mean reference gene CT value from individual CT values of corresponding target genes (ΔCT). The fold change value was calculated using the expression, where ΔΔCT represents difference between the ΔCT condition of interest and ΔCT control. The primer sets used to study the *MYB* TFs expression profile are given in the Additional file 14: Table S9.

## Abbreviations

MSU: Michigan State University; TAIR: The Arabidopsis Information Resource; PERL: Practical Extraction and Report Language; GO: Gene Ontology; BLAST: Basic Local Alignment Search Tool; MEME: Multiple Expectation Maximization for Motif Elicitation; EST: Expressed Sequence Tag; NCBI: National Center for Biotechnology Information; GEO: Gene Expression Omnibus; QRT-PCR: Quantitative Reverse Transcription Polymerase Chain Reaction.

## Competing interests

The authors declare that they have no competing interests.

## Authors’ contributions

AK performed all the bioinformatics analysis, including large-scale sequence analysis and mapping, and drafted the manuscript; SS helped in bioinformatics analysis, data mining and management; SKL conceived the idea of identification of *MYB* TF’s and designed the study; RR carried out all the wet-lab experiments; VC and KCB guided in the design of the study and drafting the manuscript. All authors read and approved the final manuscript.

## Supplementary Material

Additional file 1 **Table S1.** Nomenclature and classification of *MYB* TF family genes. Genome wide classification of *MYB* family genes including their characters such as GRAVY, PI, molecular weight and subcellular localization in rice and Arabidopsis. Click here for file

Additional file 2 **Table S2.** Functional assignment and subcellular localization of MYB TF family proteins. Molecular functional annotation of MYB TF family by gene ontology enrichment analysis including their subcellular localization in rice and Arabidopsis. Click here for file

Additional file 3 **Table S3.** Sequence alignment of intronless *MYB* genes. Sequence comparison between rice and Arabidopsis intronless genes to predict conserveness. Click here for file

Additional file 4 **Table S4.** Density of Introns. Distribution of introns in the MYB domain and other region of *MYB* genes in rice and Arabidopsis. Click here for file

Additional file 5 **Table S5.** Diurnal/circadian expression. *MYB* expression under diurnal/circadian conditions in rice and Arabidopsis. Click here for file

Additional file 6 **Table S6.** Expression of *MYB* genes. Availability of full-length complementary DNA (FL-cDNA) / expressed sequence tag (EST) consequent to *MYB* genes. Click here for file

Additional file 7 **Table S7.***MYB* regulation under abiotic stress. Expression analysis of *MYB* genes under abiotic stress conditions in rice and Arabidopsis by using publically available microarray data. Click here for file

Additional file 8 **Figure S1.***MYB* gene expression under drought stress in rice. Analysis of *MYB* gene expression under drought stress in rice. We obtained *MYB* expression from our previously published microarray gene expression experiments [64]. Click here for file

Additional file 9 **Figure S2.***MYB* gene expression under abiotic stresses in Arabidopsis. *MYB* gene expression under cold (a), drought (b) and salt (c) stresses in Arabidopsis. GENEVESTIGATOR database was used to analyze the *MYB* gene expression levels. Click here for file

Additional file 10 **Figure S3.***MYB* expression profiling using heatmap in Arabidopsis. Expression profile of *MYB* gene using heatmap for cold, drought, and salt stress, fetched by GENEVESTIGATOR database. Click here for file

Additional file 11 **Table S8.** Tissues specific *MYB* expression. Tissue-specific expression profiling of *MYB* genes in rice and Arabidopsis. Click here for file

Additional file 12 **Figure S4.***MYB* expression profiles of different tissues in rice. Tissue specific expression profile of *MYB* gene in rice examine by MSU database. Click here for file

Additional file 13 **Figure S5.** Phylogenetic analysis of MYB proteins. Phylogenetic analysis of MYB proteins in both rice and Arabidopsis. The tree was constructed by using the multiple sequence alignment of *bonafide* MYB proteins. Click here for file

Additional file 14 **Table S9.** Gene specific primers. List of gene specific primers used for QRT-PCR expression analysis of *MYB* genes in rice and Arabidopsis. Click here for file
