# Supplementary figures and images for: Genome-wide classification and expression analysis of MYB transcription factor families in rice and Arabidopsis
Source: BMC Genomics. 2012 Oct 10;13:544. doi: 10.1186/1471-2164-13-544 (PMC3542171; doi:10.1186/1471-2164-13-544)

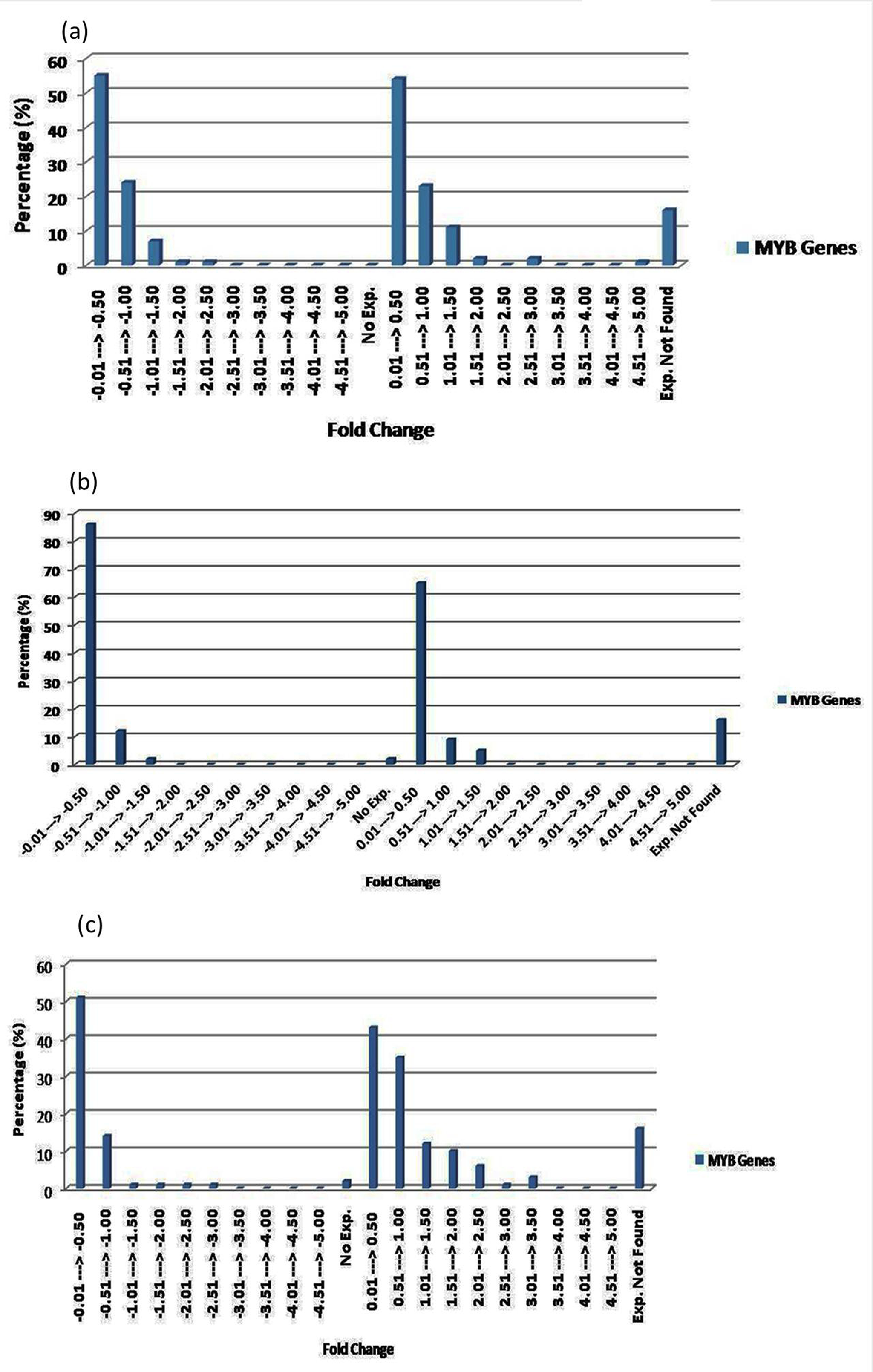

Supplement: Additional file 9 — Figure S2.MYB gene expression under abiotic stresses in Arabidopsis. MYB gene expression under cold (a), drought (b) and salt (c) stresses in Arabidopsis. GENEVESTIGATOR database was used to analyze the MYB gene expression levels. [file 1471-2164-13-544-S9.tiff]

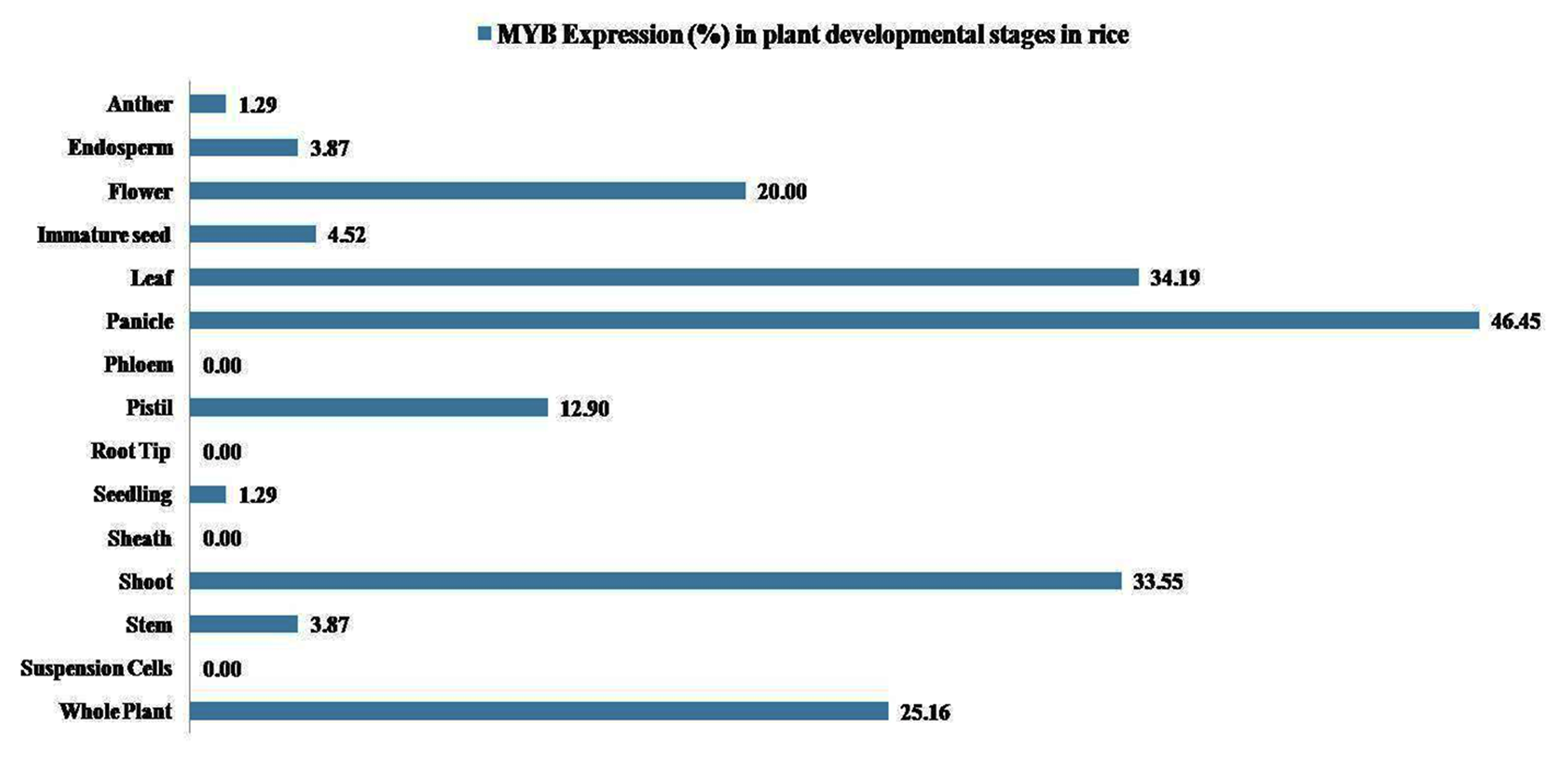

Supplement: Additional file 12 — Figure S4.MYB expression profiles of different tissues in rice. Tissue specific expression profile of MYB gene in rice examine by MSU database. [file 1471-2164-13-544-S12.tiff]

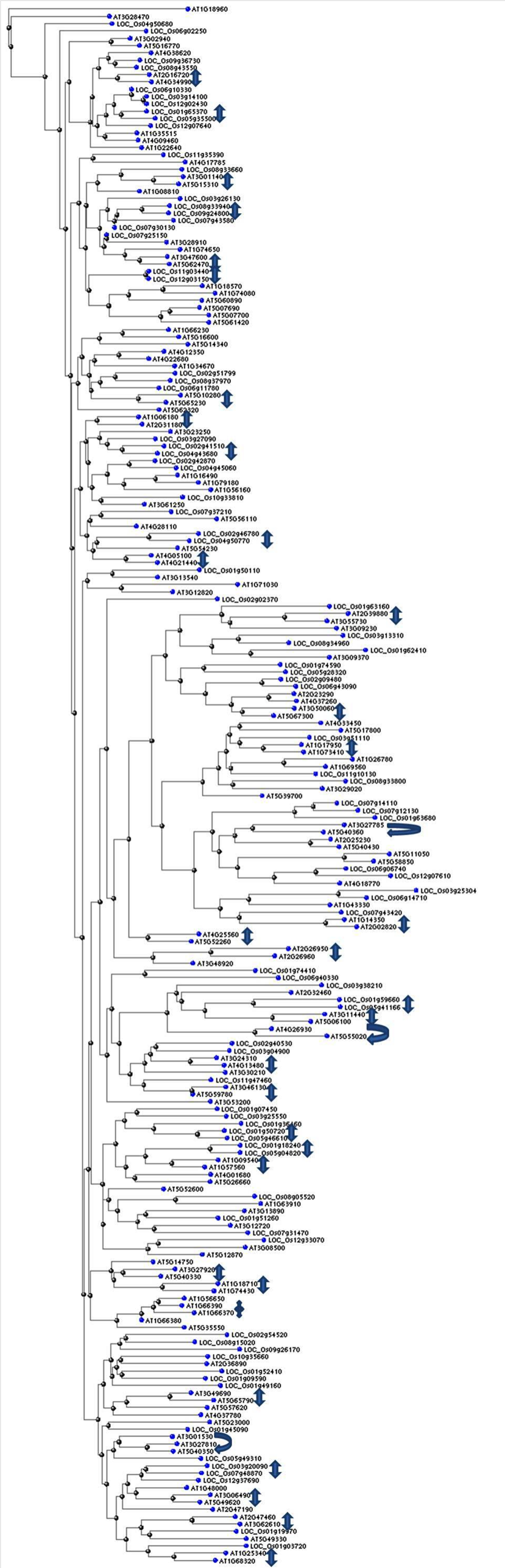

Supplement: Additional file 13 — Figure S5. Phylogenetic analysis of MYB proteins. Phylogenetic analysis of MYB proteins in both rice and Arabidopsis. The tree was constructed by using the multiple sequence alignment of bonafide MYB proteins. [file 1471-2164-13-544-S13.tiff]
